# Intra-Uterine Medication

**Published:** 1874-07

**Authors:** H. E. Woodbury


*PHILADELPHIA MEDICAL TIMES—May*, 1874:

INTRA-UTERINE MEDICATION—*By H. E. Woodbury, M.D.*—* * * My instrument, the applicator, very much resembles the injector in shape, so far as the glass tube is concerned, the only difference consisting in this, that in the applicator the openings at both terminal extremities are of the same diameter, while in the injector one of these is capillary. The edges of these openings are well rounded by heat.

Through this tube I pass a piece of steel wire about two inches longer than the tube, the temper being removed from the last two inches of this, so as to permit of its being bent to any desired curve.

The instrument is used as follows: We first moisten the end of the wire, and wrap closely around it just enough of cotton to admit of its being drawn back into the tube without difficulty.

The cotton is then dipped into fuming nitric acid, tinct. iodine, strong solution of carbolic acid, or any other fluid we may select, and withdrawn into the tube. The end of the tube is well wiped, and through the speculum introduced into the womb, dilatation of the ostium internum being sometimes necessary, in order that the tube may be passed. When the tube has entered as far as is necessary, we gently push the mop into the cavity. Thus it will be seen that our remedial agent is entirely under our control, and the cotton is not "*squeezed dry*" by coming in contact with the mucous surface of the cervix, as in Dr. Goodell's method.

